# Effects of early commercial milk supplement on the mucosal morphology, bacterial community and bacterial metabolites in jejunum of the pre- and post-weaning piglets

**DOI:** 10.5713/ajas.18.0941

**Published:** 2019-08-03

**Authors:** Ping Hu, Qingyan Niu, Yizhi Zhu, Chao Shi, Jing Wang, Weiyun Zhu

**Affiliations:** 1 National Center for International Research on Animal Gut Nutrition, Jiangsu Key Laboratory of Gastrointestinal Nutrition and Animal Health, Laboratory of Gastrointestinal Microbiology, Joint International Research Laboratory of Animal Health and Food safety, National Experimental Teaching Demonstration Center of Animal Science, College of Animal Science and Technology, Nanjing Agricultural University, Nanjing, Jiangsu, 210095, China

**Keywords:** Bacterial Community, Bacterial Metabolites, Commercial Milk Supplement, Jejunum, Mucosal Morphology, Piglets

## Abstract

**Objective:**

Sow milk (SM) may not be able to meet the piglet’s nutritional needs in late lactation. Hence, this study was conducted to investigate the effects of early commercial milk (CM) supplement on the mucosal morphology, bacterial community and bacterial metabolites in jejunum of piglets.

**Methods:**

Ten litters of newborn piglets ([Yorkshire×Landrace]×Duroc) were randomly divided into 2 groups of 5 litters. The piglets in the control group were suckled by the sow (SM), while the piglets in the treatment group (CM supplement) were supplemented with a CM supplement along with suckling from d 4 to d 28 of age.

**Results:**

No significant differences were observed about jejunal mucosal morphology on d 28 and d 35 between two groups. On d 28, the activity of lactase in the jejunum was significantly decreased in the CM group, while the activity of sucrase and the ratio of maltase to lactase were significantly increased (p<0.05). On d 35, the activity of maltase in the jejunum was significantly increased in the CM group (p<0.05), and maltase to lactase ratio tended to increase in the CM group (p = 0.065). In addition, piglets in the CM group had a higher abundance of *Clostridium XI*, *Tuicibater*, and *Moraxella* in the jejunum on d 28, while the abundance of *Lactobacillus* was significantly increased on d 35 (p<0.05).

**Conclusion:**

The early CM supplement improved the maturation of the jejunum to some extent by enhancing the maltase and sucrase activities. Moreover, the early CM supplement could help maintain the homeostasis of internal environment in jejunum by increasing the microbial-derived metabolites.

## INTRODUCTION

The small intestine (especially jejunum) is the main site of digestion and absorption, where adequate absorptive capacity depends on the maintenance of mucosal integrity constantly challenged by bacteria [1]. Although the low pH value and rapid flow of digesta in the jejunum leads to lesser numbers of bacteria compared with the hindgut, the jejunal bacterial community is important for host immunity and nutritional metabolism [2]. The jejunal beneficial bacteria could directly help inhibit pathogens colonization, or indirectly promote the jejunum epithelial cell growth and differentiation, and improve internal environment in jejunum by producing bacteria-derived metabolites, such as lactic acid and short chain fatty acids (SCFAs) [3,4]. Hence, jejunum and its microbiota are critical for maintaining intestinal homeostasis.

Breast milk is the best source of nutrients for neonatal growth and gut development [5]. Due to genetic selection and artificial rearing systems, the number of piglets delivered by sow has increased, which challenges the raising ability of sow [6]. Hence, the early commercial milk (CM) supplement could be beneficial for piglets and sows. Previous studies suggested that the early CM supplement could reduce the nutritional digestive capacity of the small intestine in piglets [7,8]. However, another study revealed that the piglets fed the CM supplement grew faster and were heavier than unsupplemented piglets [9]. While few researches are available regarding how the CM supplement affects the jejunal function and microbial community, which are highly related to the safe application of CM supplement in piglets. Therefore, we hypothesized that an early CM supplement could affect jejunal function and change the microbial community. To verity this hypothesis, jejunal morphology, disaccharidase activity, bacterial community and bacterial metabolites were investigated in this study.

## MATERIALS AND METHODS

### Animal trial

The animal experiment was approved by the Animal Experiment Committee of Nanjing Agricultural University (Nanjing, Jiangsu province, China), in accordance with the Regulations for the Administrations of Affairs Concerning the Experimental Animals (The State Science and Technology Commission of China, 1988). All experiments were performed in accordance with the approved guidelines and regulations.

Ten Yorkshire×Landrace sows were artificially inseminated by one Duroc boar. The sows had delivered on the same day. The piglets suckled the colostrum for three days after birth. On the 4th day after birth, the piglets were adjusted to 8 per litter; the average weight of piglets was 1.87±0.05 kg. Ten litters of piglets were randomly divided into the control group (sow milk, SM) and the treatment group (CM), with 5 litters in each group (n = 5). The piglets in the SM group were reared only by the sow, while the piglets in the CM group were supplemented with a CM supplement along with suckling from d 4 to d 28 of age. Piglets in the CM group had *ad libitum* access to milk supplement via a nipple system. The CM supplement was dissolved in 50°C warm water (150 g/L) and was provided via a nipple system twice a day. At 9:00 and 19:00 every day during the experimental period, we supplemented the milk supplement with a weight that is same amount of average daily feed intake (ADFI) in the nipple system, and then weighed the rest milk supplement in the nipple system when we added milk supplement next time. Then, we calculated the intake of CM supplement every day. On d 28, one piglet was randomly picked from each litter, and then slaughtered for sampling (n = 5). The remaining piglets were weaned, and the piglets in the same litter were housed in a pen, therefore each group had five pens (n = 5). All the piglets were switched to a common diet for 7 days. On d 35, one piglet was randomly picked from each litter, and then slaughtered for samples (n = 5). The piglets had free access to feed and water throughout the experimental period. The body weight (BW) of each piglet in the SM group or CM group was individually recorded on d 4, 14, 21, 28, and 35. Then, we utilized those data to calculate average daily gain (ADG) of the SM group and CM groups. The nutritional values of the milk supplement and SM are shown in Table 1 [10–13].

### Samples collection

At days 28 and 35, after fasting overnight, one piglet from each litter (5 piglets from each group, n = 5) was euthanised with sodium pentobarbital (100 mg/kg BW). The abdomen was opened and the segments (stomach, duodenum, jejunum, ileum, cecum, and colon) were identified and ligated before separation. The digesta in the jejunum was collected and mixed for measuring the pH value. Then, the digesta was immediately stored in liquid nitrogen for further analysis. Five cm jejunum tissue were fixed in 4% paraformaldehyde (100 mmol/L phosphate buffer, pH 7.4) for morphology analysis. The mucosa was isolated by gentle scraping with a glass slide, snap frozen in liquid nitrogen, and then stored in −80°C for future disaccharidase activities and gene expression analysis.

### Determination of jejunal mucosal morphology

After fixing for 24 h, formalin-fixed samples were embedded in paraffin, sectioned at a 5 μm thickness, and stained with hematoxylin and eosin for histologic examination to determine villus length, crypt depth. The villus height and crypt depth were measured and analyzed by using NIS-Elements BR software (version 2.20; Nikon, Tokyo, Japan).

### Determination of disaccharidase activities in jejunal mucosa

Each sample was homogenized in 1.0 mL cold saline mixed with protease inhibitors (Roche, Shanghai, China) on ice. Protein concentration was determined using a BCA Protein Assay Kit (Beyotime, Shanghai, China). The activity of the disaccharidase (maltase, lactase, and sucrase) was determined using commercial kits (Jiancheng Bioengineering Institute, Nanjing, China). Enzyme activity was described as U per mg of protein.

### Determination of bacterial metabolites in jejunal digesta

The concentration of lactic acid was determined using commercial kits (Jiancheng Bioengineering Institute, China). The ammonia N concentration was determined by the colorimetric method described by Nyachoti et al [14]. The concentrations of SCFAs including acetate, propionate, butyrate, valerate and branched chain fatty acids were analyzed by gas chromatography (Shimadzu GC-14B, Tokyo, Japan) [15]. A Nukol Capillary GC Column (Sigma-Aldrich, Milwaukee, WI, USA) and an FID detector were used as described by Mao et al [16]. The temperature of injector, column and detector were 110°C, 150°C, and 180°C, respectively.

### DNA extraction, polymerase chain reaction amplification and Illumina MiSeq sequencing

The total bacterial DNA was extracted from the jejunal digesta (0.1 g) according to a bead beating method described by Zoetendal et al [17]. The concentration of extracted DNA was determined using a Nano-Drop 1000 spectrophotometer (Thermo Scientific Inc., Wilmington, DE, USA). To analyze the taxonomic composition of bacterial community in the jejunal digesta, universal primers 319F (ACTCCTACGGGA GGCAGCAG) and 806R (GGACTACHVGGGTWTCTAAT) targeting the V3–V4 region of the 16S rRNA gene were chosen for the amplification and subsequent pyrosequencing of the polymerase chain reaction (PCR) products. The PCR amplification was performed using the following program: initial denaturation step at 95°C for 2 min, followed by 25 cycles at 95°C for 30 s, 55°C for 30 s, and 72°C for 30 s and a final extension at 72°C for 5 min. All amplicons were visualized on 2% agarose gels and were purified using the Axyprep DNA Gel Extraction Kit (Axygen Biosciences, Union City, CA, USA) following the recommended instructions and were quantified using Quantifluor TM-ST (Promega, Madison, WI, USA). The purified amplicons were paired-end sequenced on an Illumia Miseq platform according to a standard protocol [18].

### Bioinformatics analysis

Raw fastq files were de-multiplexed and quality-filtered using QIIME (version 1.70, CU-Boulder, CO, USA) with standard criteria as described by Sun et al [19]. Operational taxonomic units (OTUs) were clustered with a 97% similarity cut-off using UPARSE (version 7.1 http://drive5.com/uparse/), and the chimeric sequences were identified and removed using UCHIME. The bacterial diversity was assessed using the observed species, Simpson, Chao1 and Shannon indices.

### Statistical analysis

The raw sequencing data were submitted to the Sequence Read Archive under accession number SRP107026. The data were analyzed with SPSS 20.0 and shown as the means with standard error of the mean. The student’s t-test and Mann-Whitney U test were used to assess the differences between treatments, with the litter as the experimental unit for growth performance, and the piglet as the experimental unit for other indices (n = 5). The normality of the distribution of variables was tested by the Shapiro-Wilk test. The variables that had a non-normal distribution were analyzed using the nonparametric methods. The t-test and the Mann-Whitney U test were used to analyze the data that had a normal (the data of jejunal morphology, disaccharidase activities and the gene expression) or non-normal distribution (microbial data) on d 28 and d 35, respectively. Significant differences were declared when p<0.05, while p values between 0.05 and 0.10 were considered to be a tendency.

## RESULTS

### The jejunal mucosal morphology

The villus height, crypt depth and villus height to crypt depth ratio are shown in Table 2. On d 28 and d 35 of age, no significant difference in villus height, crypt depth and villus height to crypt depth ratio was observed between the CM group and the SM group.

### The jejunal mucosal disaccharidase activities and the gene expression

**Figure 1:**
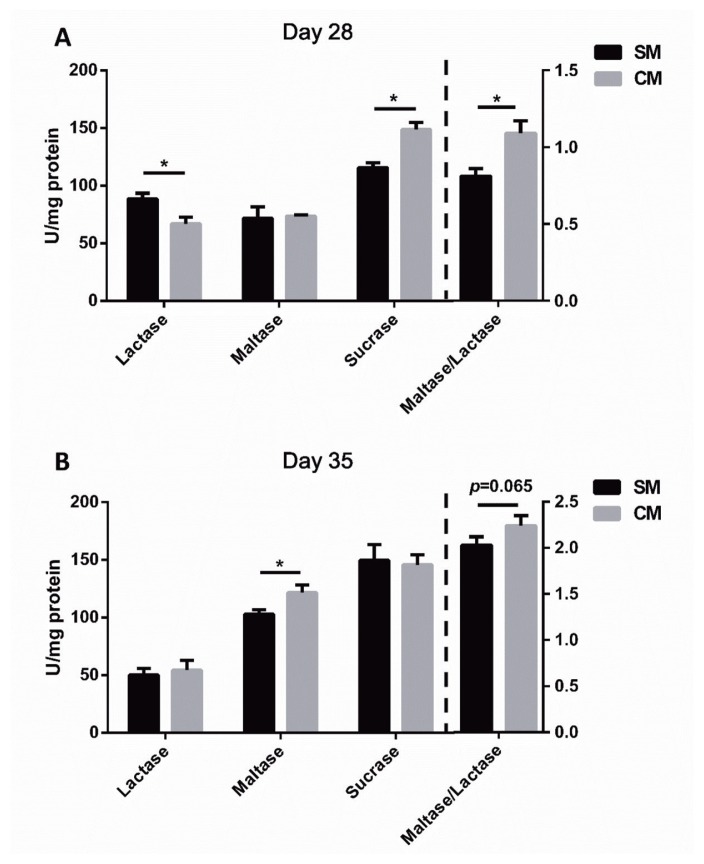
Effects of early commercial milk supplement on disaccharidase activities in the jejunum of piglets on day 28 and day 35. (A) Disaccharidase activities on day 28, (B) Disaccharidase activities on day 35. Values are means, with standard errors represented by vertical bars, n = 5. * Means are different (p<0.05) between the SM group and CM group. The SM group are the piglets reared by the sows, the CM group are the piglets supplemented with a commercial milk along with suckling.

The disaccharidase activities in jejunal mucosa of piglets are presented in Figure 1. On d 28 of age (Figure 1A), the activity of lactase in the jejunum was significantly decreased in the CM group, while the activity of sucrase and maltase to lactase ratio were significantly increased (p<0.05). On d 35 of age, the activity of maltase in the jejunum was significantly increased in the CM group (p<0.05), and maltase to lactase ratio tended to increase in the CM group (p = 0.065, Figure 1B).

### Microbial metabolites in jejunal digesta

The bacterial metabolites in jejunal digesta are presented in Table 3 and 4. On d 28 (Table 3), the concentrations of acetate and the total SCFAs were significantly increased in the jejunal digesta in the CM group (p<0.05). Piglets in the CM group had increased lactic acid concentration (p = 0.07). On d 35 of age (Table 4), the pH value significantly decreased in the jejunal digesta, and the concentration of lactic acid in the jejunal digesta statistically increased in the CM group (p = 0.04). Moreover, no obvious difference was observed in the concentrations of ammonia N and total SCFAs in the jejunal digesta of the piglets in the SM group and CM group.

### Data acquisition by Miseq sequencing and bacterial diversity in jejunal digesta

In this study, the number of average raw sequences detected in each group was at least 52,629 reads with 51,369 valid sequences (Supplementary Table S1). The overall number of OTUs detected was 17,756 at least in each sample, based on a 97% sequence similarity between reads. The rarefaction curves (Supplementary Figure S1) were generated by MOTHUR via plotting the number of reads versus the number of observed species and tended to approach the saturation plateau. The curves show that the sequencing amount was sufficient to evaluate the bacterial community in both groups. As shown in Supplementary Table S2, on d 28 of age, no statistical difference was observed in the diversity indices (Simpson) and richness estimators (observed species and Chao 1) of jejunal bacteria between SM group and CM group (p>0.05). However, compared with the SM group, the indices (Shannon) tended to increase in the CM group (p = 0.07). On d 35, no statistical difference was observed in the diversity indices (Shannon and Simpson) and richness estimators (observed species and Chao 1) of jejunal bacteria between the SM group and the CM group.

### Bacterial community determined by MiSeq sequencing

**Figure 2:**
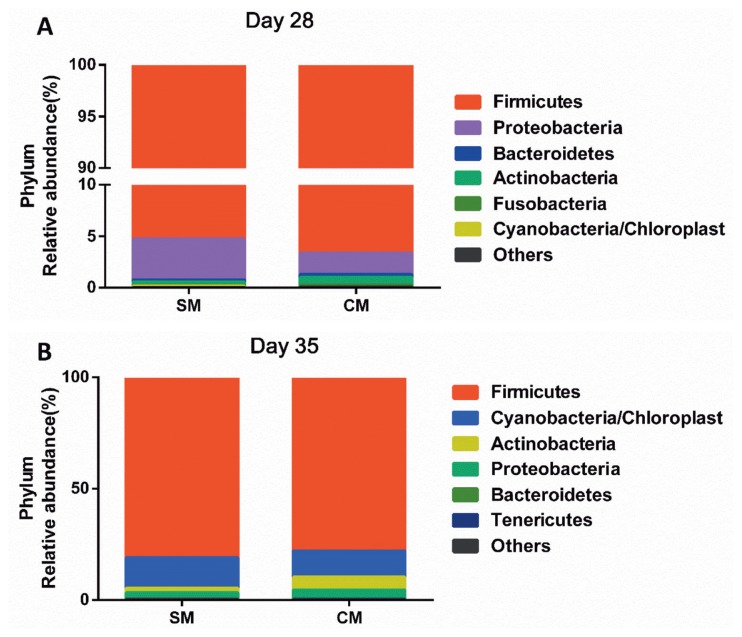
Relative abundance of microbiota at the phylum level. (A) On day 28; (B) On day 35. Values are means, n = 5. The SM group are the piglets reared by the sows, the CM group are the piglets supplemented with a commercial milk supplement along with suckling.

The bacterial community was assessed at different taxonomic levels. At the phylum level, as shown in Figure 2, the Firmicutes was the predominant phylum in all samples by d 28 of age, accounting for 95.97% of the total 16S rRNA gene sequences. Proteobacteria was the second dominant phylum, represented by 3.00% of total 16S rRNA gene sequences, followed by the bacteria from phyla Bacteroidetes (0.25%), and Actinobacteria (0.54%). By d 35 of age, the Firmicutes was the predominant phylum in all samples, accounting for 77.97% of the total 16S rRNA gene sequences. Cyanobacteria/Chloroplast was the second dominant phylum, represented by 11.78% of total 16S rRNA gene sequences, followed by the bacteria from phyla Actinobacteria (5.72%), Proteobacteria (4.09%). No statistical difference in the bacterial community at the phylum level was observed between the SM group and the CM group.

**Figure 3:**
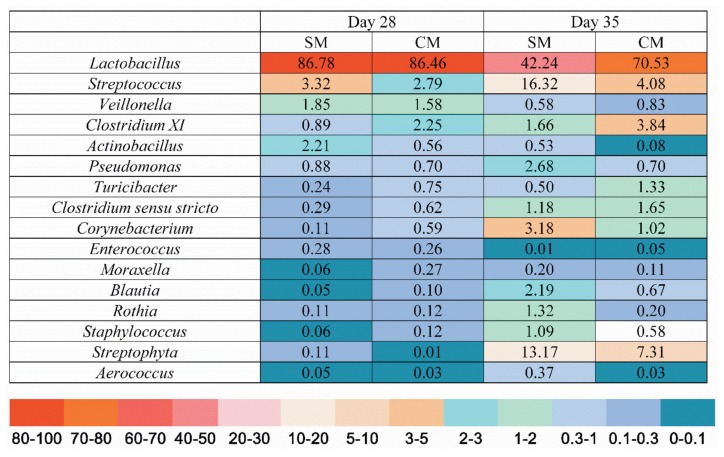
Relative abundance of microbiota in jejunal digesta at the genus level on day 28 and day 35. Values are means, n = 5.

**Figure 4:**
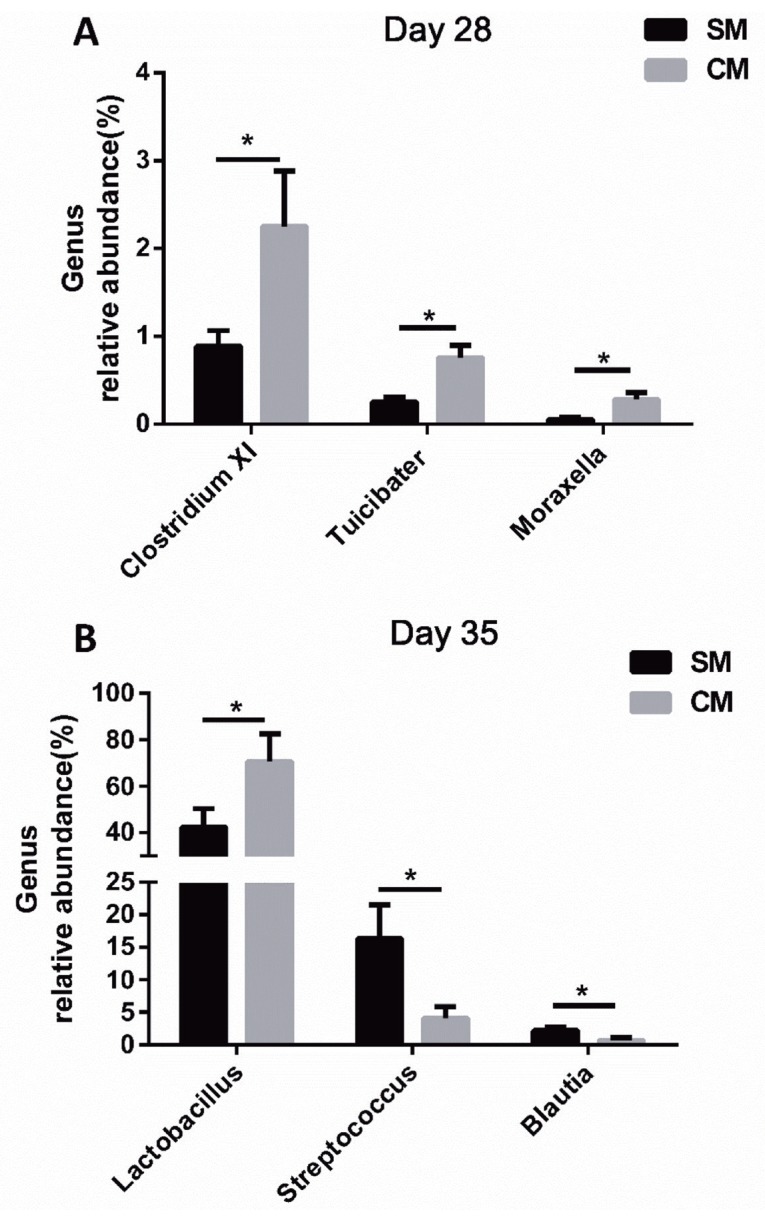
Effects of early commercial milk supplement on the significantly changed genera in jejunal digesta. (A) On day 28; (B) On day 35; values are means, with standard errors represented by vertical bars, n = 5. * Means are different (p<0.05) between the SM group and CM group. The SM group are the piglets reared by the sows, the CM group are the piglets supplemented with a commercial milk supplement along with suckling.

At the genus level, as shown in Figure 3, by d 28 of age, the predominant genera were the *Lactobacillus*, *Streptococcus*, *Veillonella*, and *Clostridium XI*, while, there was no statistical difference between the SM group and the CM group (p>0.05). By d 28 of age (Figure 4A), piglets in the CM group had a higher abundance of *Clostridium XI*, T*uicibater*, and *Moraxella* in the jejunum than those of piglets in the SM group (p<0.05). By d 35 of age (Figure 4B), the *Lactobacillus*, *Streptophyta*, *Streptococcus*, and *Clostridium XI* were predominant. In addition, the abundance of *Lactobacillus* was significantly increased, while the abundance of *Streptococcus* and *Blautia* was statistically decreased in the CM group by d 35 of age (p<0.05).

## DISCUSSION

Previous study revealed that an early CM supplement could challenge the gut function, and damage the host health [20]. In our study, we revealed that milk supplement numerically increased the litter weight, but the difference was not significant, and no significant difference was observed in ADG and ADFI between two groups during experiment period [21]. The villus height and the crypt depth are useful criterions to evaluate the nutrient digestion and absorption capacity of the jejunum [22]. In the present study, no significant difference was observed in jejunal morphology (villus height and the crypt depth) between the SM group and CM group on d 28 and d 35 of age, indicating that the early milk supplement has no effect on the jejunal mucosa structure. It is noteworthy that disaccharidases localized on the jejunal microvilli could promote the newborn’s development by contributing to the carbohydrate digestion and absorption [23]. In the present study, the piglets in the CM group had a higher sucrase activity and lower lactase activity than those in the SM group by d 28 of age, which suggested that the CM supplement could alter the absorption of different sugars in piglets. In addition, the piglets in the CM group had higher maltase activity than those in the SM group by d 35. The maltase to lactase ratio, an index of gut maturation, was increased in the CM group by d 28 and d 35 of age, suggesting that CM supplement contributes to gut maturation [24]. Therefore, the early CM supplement not only had no negative effect on the growth performance and the jejunal morphology, but also promoted the maturity of jejunum to some extent by altering the disaccharidases activities.

The bacterial community is important to the host intestinal physiology, affecting morphology, nutrient digestion, and metabolism [25]. Early nutritional composition is the major factor influencing the bacterial community [26]. In the present study, the Firmicutes, Proteobacteria, Bacteroidetes and Actinobacteria were the predominant bacteria at the phylum level, which is consistent as reported by Cao et al [27]. At the genus level, the piglets in the CM group had a higher abundance of *Clostridium XI*, *Tuicibater*, and *Moraxella* in the jejunum than those of piglets in the SM group by d 28 of age. *Clostridium XI* belonging to the Firmicutes contributes to the increase of β-glucuronidase activity, which is conductive to nutrient utilization and gut health [28]. Moreover, the CM piglets had a higher abundance of *Lactobacillus* than the SM piglets by d 35 of age, indicating that the early CM supplement could help maintain the intestinal homeostasis by increasing the abundance of beneficial bacteria [29]. The microbial stability in the gut plays a critical role in maintaining the health status of the host gut by impacting nutrient utilization, enhancing gut barrier function and simulating immune development [30]. Thompson et al [31] revealed that the early CM supplement may shape colonization of microbiota and increase the diversity of microbiota. Consistent with the previous research, piglets in the CM group had an increased tendency of microbial diversity in the present study by d 28 of age, suggesting that the early CM supplement could make the jejunal bacterial community more stable. Therefore, the early CM supplement changed the jejunal bacterial community and benefitted the stability of the jejunal bacterial community by increasing diversity of microbiota.

The bacteria could modulate the gut physiology by ferment ing simple structure carbohydrates to produce the metabolites, such as the SCFAs and lactate, which could promote the energy intake, stimulate water and sodium absorption, and low the luminal pH value [30,32]. In our study, the increased total SCFAs (mainly the acetate) in the CM group by d 28 of age indicated that the bacterial fermentation ability was enhanced. Previous research revealed that *Clostridium XI* had the SCFA-producing ability, which may be the reason for the increasing total SCFAs in the present study [33]. Increased SCFAs could keep the homeostasis of the intestinal environment and improve the gut immune system [34]. In addition, the lactic acid concentration was statistically increased, and the pH value was significantly decreased in the CM group by day 35 of age. The increased lactic acid concentration and decreased pH value were probably caused by the increased abundance of *Lactobacillus*, as the *Lactobacillus* is the major lactic acid bacteria and the increased lactic acid leaded to the decrease of pH [29]. The low pH value in jejunum is conducive to maintain the integrity of the mucosal morphology, partially inhibit harmful bacteria, and promote the colonization of beneficial bacteria [35]. The fermentation products from these facultative anaerobes is available to the host epithelial cells as an additional energy source, and can also serve as the substrates for other microbes, such as *Veillonella* species which could utilize the lactic acid as a carbon and energy source to provide energy for the host [36]. Therefore, these results showed that the early CM supplement could help maintain a stable jejunum internal environment via producing the metabolites and decreasing the pH value.

## CONCLUSION

The early CM supplement had no effect on the jejunal mucosal morphology in piglets but improved the maturation of the jejunum to some extent by enhancing the maltase and sucrase activities. Moreover, the early CM supplement induced a more stable bacterial community by increasing the diversity indices with changing the bacterial composition. Finally, the early CM supplement promoted the homeostasis of internal environment in jejunum by increasing the microbial-derived metabolites, such as acetate and lactate.

## Supplementary Data



## Figures and Tables

**Table 1:** The nutrient composition of the commercial milk supplement (air-dry basis) and sow milk

Items	Milk supplement	Sow milk1)
Ingredient (%)		
Corn	39.20	-
Whey powder	18.95	-
Soybean meal	18.43	-
Extruded soybean	15.38	-
Fish meal	6.23	-
Dicalcium phosphate	0.38	-
Sodium chloride	0.50	-
L-lysine	0.50	-
DL-methionine	0.30	-
Zinc chloride	0.10	-
Vitamins	0.03	-
Phytase	0.01	-
Calculated composition (%)		
Crude protein	20.63	5.31 to 15.33 [10]
Crude fibre	1.64	-
Crude ash	5.67	0.89 to 1.20 [11]
Calcium	0.76	0.07 to 0.17 [12]
Phosphorus	0.64	0.11 to 0.13 [12]
NaCl	0.60	-
Lysine	1.50	0.35 to 1.05 [13]
Moisture	6.90	-

1) Data from other studies.

**Table 2:** Effects of the early commercial milk supplement on the mucosal morphology in jejunum of piglets on the day 28 and 35

Items	Treatments1)		SEM	p-value

	SM	CM		
day 28				
Villus height (μm)	321.34	307.85	26.09	0.786
Crypt depth (μm)	105.34	96.16	9.79	0.407
Villus height:crypt depth (%)	3.02	3.33	0.45	0.592
day 35				
Villus height (μm)	259.01	274.83	29.27	0.611
Crypt depth (μm)	91.06	97.47	10.74	0.568
Villus height:crypt depth (%)	2.87	2.83	0.21	0.833

Values are means of 5 replicates per treatment.

SEM, standard error of mean.

1) The SM group are the piglets reared by the sows. The CM group are the piglets supplemented with a commercial milk replacer along with suckling.

**Table 3:** The pH value and the concentrations of the lactic acid, ammonia N, different SCFAs and total SCFAs in the jejunal digesta of piglets on day 28

Items	Treatments1)		SEM	p-value

	SM	CM		
pH value	5.89	5.97	0.04	0.251
Acetate (μmol/g)	0.30	0.56	0.06	0.004
Propionate (μmol/g)	0.11	0.14	0.04	0.528
Butyrate (μmol/g)	0.05	0.06	0.01	0.932
Valerate (μmol/g)	0.07	0.09	0.01	0.267
BCFAs (μmol/g)2)	0.04	0.05	0.01	0.334
Total SCFAs (μmol/g)3)	0.58	0.90	0.09	0.011
Ammonia N (μmol/g)	28.17	21.54	5.64	0.314
Lactic acid (mmol/L)	1.14	1.39	0.13	0.072

Values are means of 5 replicates per treatment.

SCFAs, short chain fatty acids; SEM, standard error of mean; BCFAs, branched chain fatty acids.

1) The SM group are the piglets reared by the sows. The CM group are the piglets supplemented with a commercial milk replacer along with suckling.

2) Sum of isobutyrate and isovalerate.

3) Sum of acetate, propionate, butyrate, valerate, isobutyrate, and isovalerate.

**Table 4:** The pH value and the concentrations of the lactic acid, ammonia N, different SCFAs and total SCFAs in the jejunal digesta of piglets on day 35

Items	Treatments1)		SEM	p-value

	SM	CM		
pH value	6.33	5.79	0.15	0.032
Acetate (μmol/g)	0.41	0.57	0.11	0.258
Propionate (μmol/g)	0.09	0.15	0.06	0.317
Butyrate (μmol/g)	0.05	0.05	0.04	0.944
Valerate (μmol/g)	0.09	0.07	0.03	0.713
BCFAs (μmol/g)2)	0.06	0.05	0.02	0.676
Total SCFAs (μmol/g)3)	0.69	0.89	0.24	0.448
Ammonia N (μmol/g)	16.62	13.94	5.88	0.664
Lactic acid (mmol/L)	1.02	1.31	0.12	0.041

Values are means of 5 replicates per treatment.

SCFAs, short chain fatty acids; SEM, standard error of mean; BCFAs, branched chain fatty acids.

1) The SM group are the piglets reared by the sows. The CM group are the piglets supplemented with a commercial milk replacer along with suckling.

2) Sum of isobutyrate and isovaleric;

3) Sum of acetate, propionate, butyrate, valerate, isobutyrate, and isovalerate.
