# Supplementary material for: Effects of early commercial milk supplement on the mucosal morphology, bacterial community and bacterial metabolites in jejunum of the pre- and post-weaning piglets
Source: Asian-Australas J Anim Sci. 2019 Aug 3;33(3):480–9. doi: 10.5713/ajas.18.0941 (PMC7054622; doi:10.5713/ajas.18.0941)
Supplement: Supplementary file 1 [file ajas-18-0941-suppl.pdf]

Supplementary Table S1. The raw sequences and valid sequences

	Raw Data		Valid Data		Valid%	Q20%	Q30%	GC%
Sample	Tag	Base	Tag	Base				
Day 28								
SM.	69378.6	32.886M	63795.2	26.996M	95.804	95.570	86.638	51.504
MR.	78723.8	37.314M	76927.8	32.542M	97.512	95.246	85.814	51.368
Day 35								
SM.	53663.0	25.436M	52511.2	21.976M	97.794	95.468	86.306	52.176
MR.	52629.2	24.946M	51369.4	21.302M	97.702	95.304	85.864	52.540

Supplementary Table S2. Diversity estimation of the 16S rRNA gene libraries from bacteria in the jejunal digesta of the piglets on the 28th and 35th day

Items	SM	MR	P-value
28 day			
observed_species	1685.80±191.61	2157.40±271.97	0.19
Shannon	3.92±0.19	5.17±0.52	0.07
Simpson	0.74±0.03	0.84±0.05	0.16
Chao1	18901.07±2687.47	22122.90±3993.06	0.52
35 day			
observed_species	1478.0±205.83	1529.80±76.30	0.82
Shannon	4.43±0.59	5.09±0.38	0.38
Simpson	0.81±0.06	0.87±0.03	0.34
Chao1	15424.36±1351.22	12976.43±1884.33	0.32

The row with different letters means significant difference ($P < 0.05$); no letters means no significant difference ($P \geq 0.05$). SM showed that the piglets were reared by the sows. MR showed that the piglets were supplemented with a commercial milk supplement along with suckling. n = 5 per group per day.

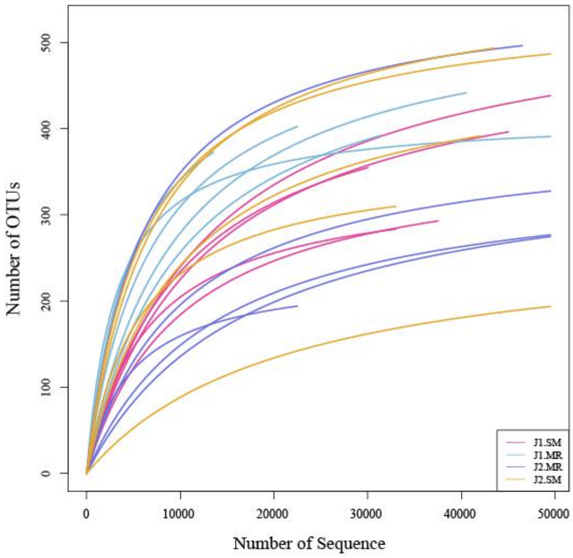

Supplementary Figure S1. Rarefaction curves comparing the number of sequences with the number of observed species found in the 16S rRNA gene libraries from bacteria in the jejunal digesta. J1.SM (—) showed that the piglets were reared by the sows on the 28th day; J1.MR (—) showed that the piglets were supplemented with a commercial milk supplement along with suckling on the 28th day; J2.SM (—) showed that the piglets were reared by the sows on the 35th day; J2.MR (—) showed that the piglets were supplemented with a commercial milk supplement along with suckling on the 28th day on the 35th day. n = 5 per group per day.
